# Benefit of replanning in MR-guided online adaptive radiation therapy in the treatment of liver metastasis

**DOI:** 10.1186/s13014-021-01813-6

**Published:** 2021-05-04

**Authors:** Michael Mayinger, Roman Ludwig, Sebastian M. Christ, Riccardo Dal Bello, Alex Ryu, Nienke Weitkamp, Matea Pavic, Helena Garcia Schüler, Lotte Wilke, Matthias Guckenberger, Jan Unkelbach, Stephanie Tanadini-Lang, Nicolaus Andratschke

**Affiliations:** Department of Radiation Oncology, University Hospital of Zurich, University of Zurich, Raemistrasse 100, 8091 Zurich, Germany

## Abstract

**Purpose:**

To assess the effects of daily adaptive MR-guided replanning in stereotactic body radiation therapy (SBRT) of liver metastases based on a patient individual longitudinal dosimetric analysis.

**Methods:**

Fifteen patients assigned to SBRT for oligometastatic liver metastases underwent daily MR-guided target localization and on-table treatment plan re-optimization. Gross tumor volume (GTV) and organs at risk (OARs) were adapted to the anatomy-of-the-day. A reoptimized plan (RP) and a rigidly shifted baseline plan (sBP) without re-optimization were generated for each fraction. After extraction of DVH parameters for GTV, planning target volume (PTV), and OARs (stomach, duodenum, bowel, liver, heart) plans were compared on a per-patient basis.

**Results:**

Median pre-treatment GTV and PTV were 14.9 cc (interquartile range (IQR): 7.7–32.9) and 62.7 cc (IQR: 42.4–105.5) respectively. SBRT with RP improved PTV coverage (V100%) for 47/75 of the fractions and reduced doses to the most proximal OARs (D1cc, Dmean) in 33/75 fractions compared to sBP. RP significantly improved PTV coverage (V100%) for metastases within close proximity to an OAR by 4.0% (≤ 0.2 cm distance from the edge of the PTV to the edge of the OAR; n = 7; *p* = 0.01), but only by 0.2% for metastases farther away from OAR (> 2 cm distance; n = 7; *p* = 0.37). No acute grade 3 treatment-related toxicities were observed.

**Conclusions:**

MR-guided online replanning SBRT improved target coverage and OAR sparing for liver metastases with a distance from the edge of the PTV to the nearest luminal OAR < 2 cm. Only marginal improvements in target coverage were observed for target distant to critical OARs, indicating that these patients do not benefit from daily adaptive replanning.

**Supplementary Information:**

The online version contains supplementary material available at 10.1186/s13014-021-01813-6.

## Introduction

The implementation of stereotactic body radiation therapy (SBRT) was an important milestone in local treatment for oligometastatic and medically inoperable cancers [1, 2]. High rates of local control in various disease sites including hepatic metastases have been observed, as long as high biologically effective (BED) doses could be delivered [3–5].

SBRT requires maximum accuracy in treatment delivery to ensure that the high irradiation doses are precisely administered to the target structures while simultaneously sparing surrounding normal tissues. Especially when treating abdominal malignancies, such as liver metastases, the dose of SBRT is often limited by the proximity of gastrointestinal organs [6, 7] and PTV compromises are necessary to minimize the risk of radiation-induced gastrointestinal toxicity. This may translate in reduced local control if a minimum BED of 100 Gy cannot be achieved [8–10]. Both intra-fraction respiratory motion and physiologic organ alterations have been identified as critical factors influencing treatment accuracy [11, 12].

Cone-beam based image-guided radiation therapy (IGRT) strategies have substantially improved the accuracy of SBRT in liver SBRT [10]. However, low soft tissue contrast combined with slow image acquisition relative to breathing motion do not allow accurate visualization of the hepatic metastases themselves and upper abdominal organs at risk. Therefore, stereotactic MR-guided online adaptive radiation therapy (SMART) has been suggested to overcome the limitations of low soft-tissue contrast IGRT by combining daily MR based treatment adaptation and replanning with MR based target localization and continuous real-time tracking of the moving target.

The feasibility of SMART was shown in a prospective trial demonstrating improved PTV coverage and/or simultaneous organs at risk (OARs) sparing for abdominal malignancies [13]. While the advantage of MR-guided imaging and gating has been well established, the benefit of daily on-table adaptive replanning, a time- and resource-intense process, for different locations of hepatic metastases remains uncertain.

The aim of this study therefore was to quantify a dosimetric benefit of online replanning on top of MR-guided setup correction and gating for liver metastases and derive recommendations when a SMART approach is mandatory or can be safely omitted.

Methods.

### Patient characteristics

All patients treated with magnetic resonance image guided radiation therapy (MRgRT) for liver metastases at the Radiation Oncology Department of the University Hospital Zurich between 04/2019 and 04/2020 were identified from our institutional SBRT database (Table 1). A total of 15 patients with oligometastatic liver metastases were identified that underwent MR-guided SBRT/SMART. Patient characteristics are summarized in Table 1. Two patients had received prior liver SBRT on a C-arm Linac. One patient presented with a local recurrence at a previously irradiated location, while the other patient presented with a newly developed hepatic metastasis. Median follow-up was 8 months (range 3–14). The fractionation scheme used was based on the treating physician’s decision, with the most common fractionation scheme used being 5 × 9 Gy to the 65% isodose (10/15 patients; Table 2). This analysis was approved by the cantonal ethics committee Zurich (BASEC-Nr. 2018-01794) and conducted in accordance with the ethical standards of the 1964 Declaration of Helsinki and its later amendments [14]. All patients gave their consent for retrospective data analysis.

**Table 1:** Patient characteristics

	All patients (n = 15)
Sex	
Male	12
Female	3
Age at time of SABR, median (range)	46 (32–63)
Performance status (ECOG)	0 (0–1)
Previous liver irradiation	2
GTV mean, SD [cc]	29.4 ± 33.1
PTV mean, SD [cc]	92.1 ± 77.5
Primary tumor	
Gastrointestinal	9
Breast	2
Melanoma	2
NSCLC	1
Bladder	1

**Table 2:** Distance to the closest organ at risk and prescription dose for each patient

Patient	Organ	Distance (edge PTV to OAR)	Prescription dose
A	Heart	0.1 cm	5 × 9 Gy @ 65%
B	Heart	0.2 cm	5 × 9 Gy @ 65%
C	Heart	0.6 cm	5 × 9 Gy @ 65%
D	Bowel	Overlap	5 × 5 Gy @ 65%
E	Bowel	2.5 cm	4 × 9 Gy @ 65%
F	Heart	4.5 cm	5 × 9 Gy @ 65%
G	Stomach	3.0 cm	5 × 9 Gy @ 65%
H	Heart	Overlap	5 × 9 Gy @ 65%
I	Heart	0.2 cm	5 × 8 Gy @ 65%
J	Heart	7.0 cm	5 × 9 Gy @ 65%
K	Bowel	8.0 cm	6 × 5 Gy @ 80%
L	Bowel	Overlap	5 × 9 Gy @ 65%
M	Bowel	3.0 cm	5 × 7 Gy @ 80%
N	Stomach	Overlap	5 × 6 Gy @ 65%
O	Heart	4.0 cm	5 × 9 Gy @ 65 %

### Simulation and initial treatment planning

Before undergoing MRI simulation, all patients were thoroughly checked for eligibility, including their ability to perform a 30-second expiration breath-hold. MR simulation was performed on the MRIdian system (ViewRay, Sunnyvale, CA) with testing of gross tumor volume (GTV) tracking in sagittal cine MR-imaging. Patients then underwent a 3D inspiratory-breath hold planning CT scan with intravenous contrast agent, which was deformably registered to the 3DMR scan to obtain electron density data. GTV and OARs were manually delineated by the treating physician. A clinical target volume (CTV) was created by expanding the GTV by an isotropic margin of 0.5 cm and cropping at the boundary of the liver. Planning target volume (PTV) was generated by an isotropic 0.5 cm expansion of the CTV. A Monte Carlo algorithm based, intensity-modulated RT (IMRT) step and shoot treatment plan, referred to as “baseline plan (BP)” was calculated, using a grid spacing of 0.2 cm. The IMRT plans included 9 to 11 beams, avoiding entrance dose in the contralateral side. Ring structures around the PTV were created to optimize conformity. A planning objective limiting the D0.1 cc of the PTV to be at maximum 156% of the dose prescribed was given (e.g. 70.2 Gy for the most commonly used 45 Gy). The planning objectives for the GTV were V95% ≥ 135% of the dose prescribed and D0.1 cc ≥  152%. For bowel, stomach and duodenum, in-house dose-volume constraints were enforced (D1cc < 26 Gy in 5 fractions). If necessary, PTV coverage was compromised to fulfill these constraints. In this case, a compromised PTV (CTV) was created with a pullback of 0.3 cm (0.6 cm) from the OAR. The prescribed dose was delivered to the compromised PTV (CTV) and a dose below the OAR constraint was delivered to the remaining PTV. All plans were normalized to achieve V100% of the PTV (or the compromised PTV) greater or equal to 95%. No dose constraint was enforced for the heart. If all the constraints were respected without any PTV compromise, no further reduction of the OAR dose was attempted, but rather an increase of the plan conformity by minimizing the dose to the ring structures.

### Online plan reoptimization and treatment delivery

**Fig. 1:**
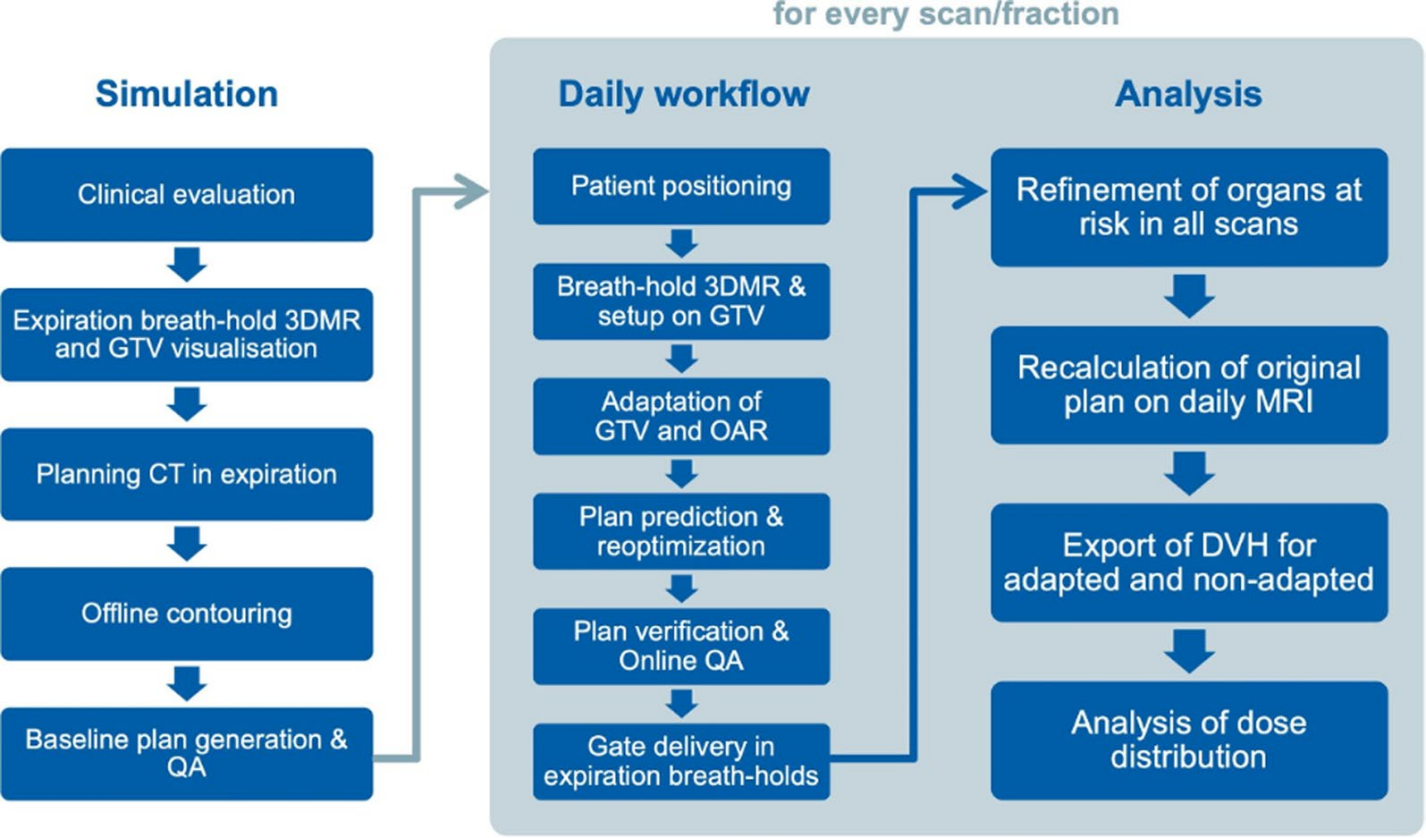
Comprehensive SMART analysis workflow consisting of the simulation and treatment planning, daily adaptive MRgRT workflow and retrospective analysis steps for DVH generation based on the anatomy-of-the day

The employed comprehensive SMART analysis workflow consisting of the computed tomography (CT) and MRI simulation, daily MR-guided adaptive replanning (MRgRT) including weight or full optimization, and subsequent analysis is illustrated in Fig. 1. Details of the SMART workflow [15] and the dose-volume histogram (DVH) analysis have been published previously [12].

Patients underwent daily MR-guided set-up and on-table treatment plan re-optimization for each fraction (n = 75). The GTV as well as the OARs were recontoured and adapted to the anatomy-of-the-day within the volume of 2 cm isotropic expansion of the PTV. In a first step, we performed a weight optimization for each patient, which consists in keeping the MLC leafs in the same positions as the baseline plan and reoptimizing the monitor units delivered by each segment. If the PTV coverage was the same or better compared to the original plan and the OARs constraints were all respected, the weight-optimized plan was delivered for this fraction. If not, a full plan optimization was performed, i.e. the MLC leaves positions were optimized based on the adapted structures. Based on the physician decision, one of the two was delivered as the reoptimized plan of the day (RP). Gated expiration breath-hold treatment delivery was performed under continuous sagittal MR guidance.

### Treatment plans

Multiple plans were calculated for each patient in the clinical routine and for this planning study: The BP was prepared on the simulation scans, approved by the treating physician, never delivered but used as a starting point for the daily adaptations and creation of the RP as described in the previous subsection. For this retrospective data analysis, the BP was also copied on the daily anatomies, rigidly shifted to achieve optimal target coverage and recalculated obtaining the shifted baseline plan (sBP). In the data analysis we compared the BP, sBP and RP to quantify the benefit of the reoptimization.

### Analysis of treatment plans

For detailed DVH analysis, all OAR were fully contoured in every individual MR scan used for treatment delivery. All reference plans and clinically delivered reoptimized plans were exported from the Viewray system (Oakwood Village, OH, USA) and imported into Eclipse Treatment Planning System (version 13.0, Varian Medical Systems, Palo Alto, CA, USA). DVH parameters were evaluated for GTV, PTV (V100%, D95%), and OARs (Dmax; D1cc). The dose was evaluated on the daily MR image, while no renormalization was performed. The detailed python notebook including all steps of analysis, data and plots is available under https://github.com/rmnldwg/liver-smart.

### Statistical analysis

Shapiro-Wilk test was computed to ensure the assumption of normality was not violated in the data. Statistical analysis of dosimetric parameters was performed using an unpaired t-test for comparing the ≤ 0.2 cm versus the > 2 cm groups, while a paired t-test was employed to compare sBP to RP for each patient (GraphPad Prism version 7.00 for MAC, GraphPad Software, La Jolla California USA). A *p *value below 0.05 was considered to be statistically significant.

## Results

### Treatment planning and adaptation

In total, 75 fractions were delivered. Full optimization was performed for 51 fractions. For the remaining 24 fractions, no full optimization was performed, and only weight-optimized plans were delivered. A full optimization for each fraction was carried out in 6 patients and at least one full optimization over the course of therapy was performed in 13 out of 15 patients.

### Interfractional changes in tumor volumes

Median pre-treatment GTV volume was 14.9 cc (interquartile range (IQR): 7.7–32.9) and PTV volume was 62.7 cc (IQR: 42.4–105.5). Median GTV and PTV changes compared to baseline were 0 cc (IQR: − 0.6 to 0) and 0.4 cc (IQR: 0–2.5) respectively. The volume of the GTV was not adjusted from the baseline plan in 34% of all fractions. Detailed data of adaptive volume changes are shown in the Additional file 1.

### Impact of plan adaptation

Mean conformity index was 1.14 for RP and 1.12 for sBP (range 0.95–1.24 vs. 0.94–1.29). Mean dose in 700 cc of the liver was also similar for RP and sBP (9.25 Gy vs. 9.24 Gy).

**Fig. 2:**
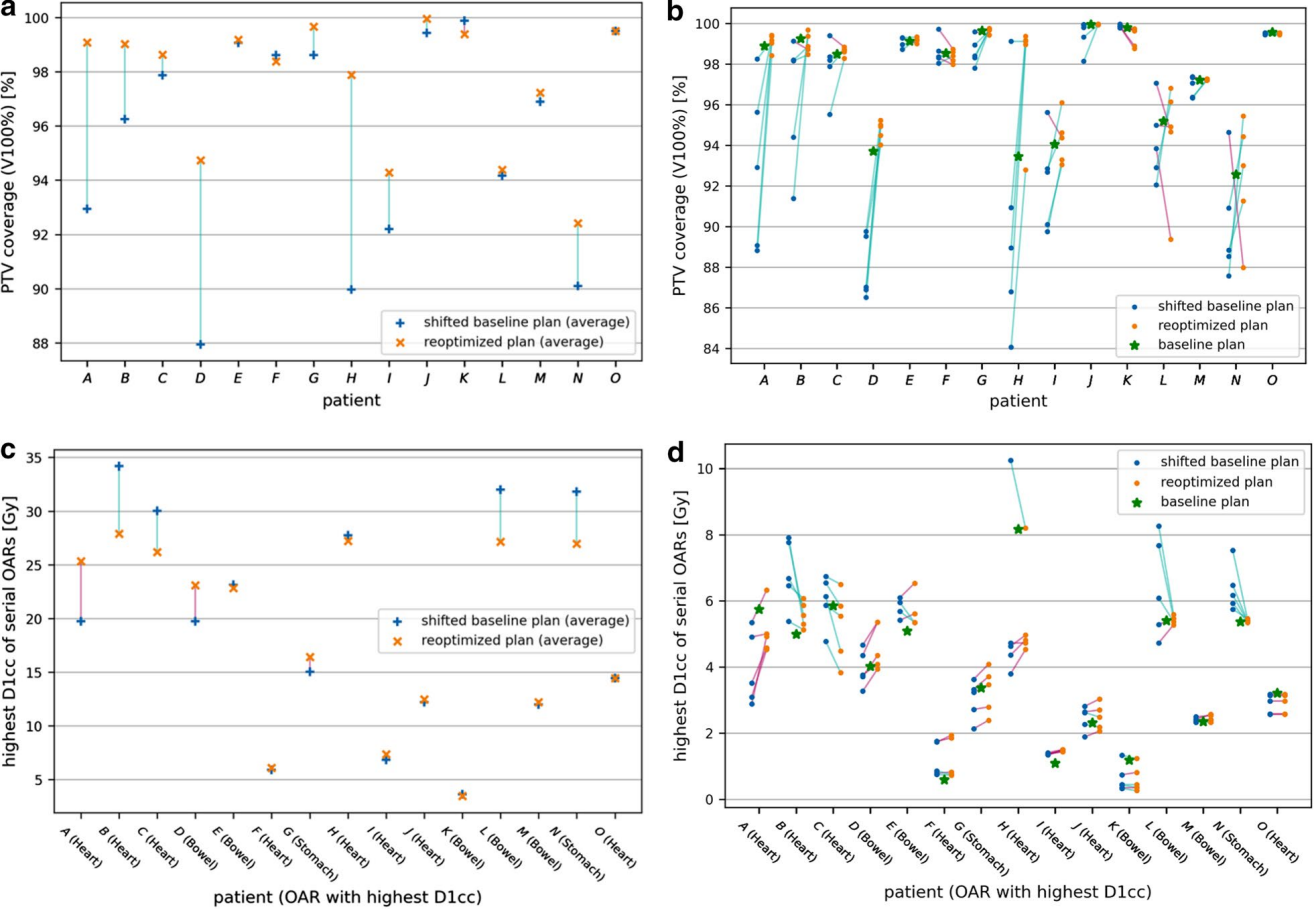
**a**, **b** PTV coverage (V100 %) for each patient comparing baseline plans (BP), rigidly shifted baseline plans (sBP), and reoptimized treatment plans (RP), averaged over all fractions (**a**) and for each individual fraction (**b**). **c**, **d** D1cc in Gy for the OAR receiving the highest dose (indicated for each patient), averaged over all fractions (**c**) and for each individual fraction (**d**). The distance to OARs is shown in Table 2

**Fig. 3:**
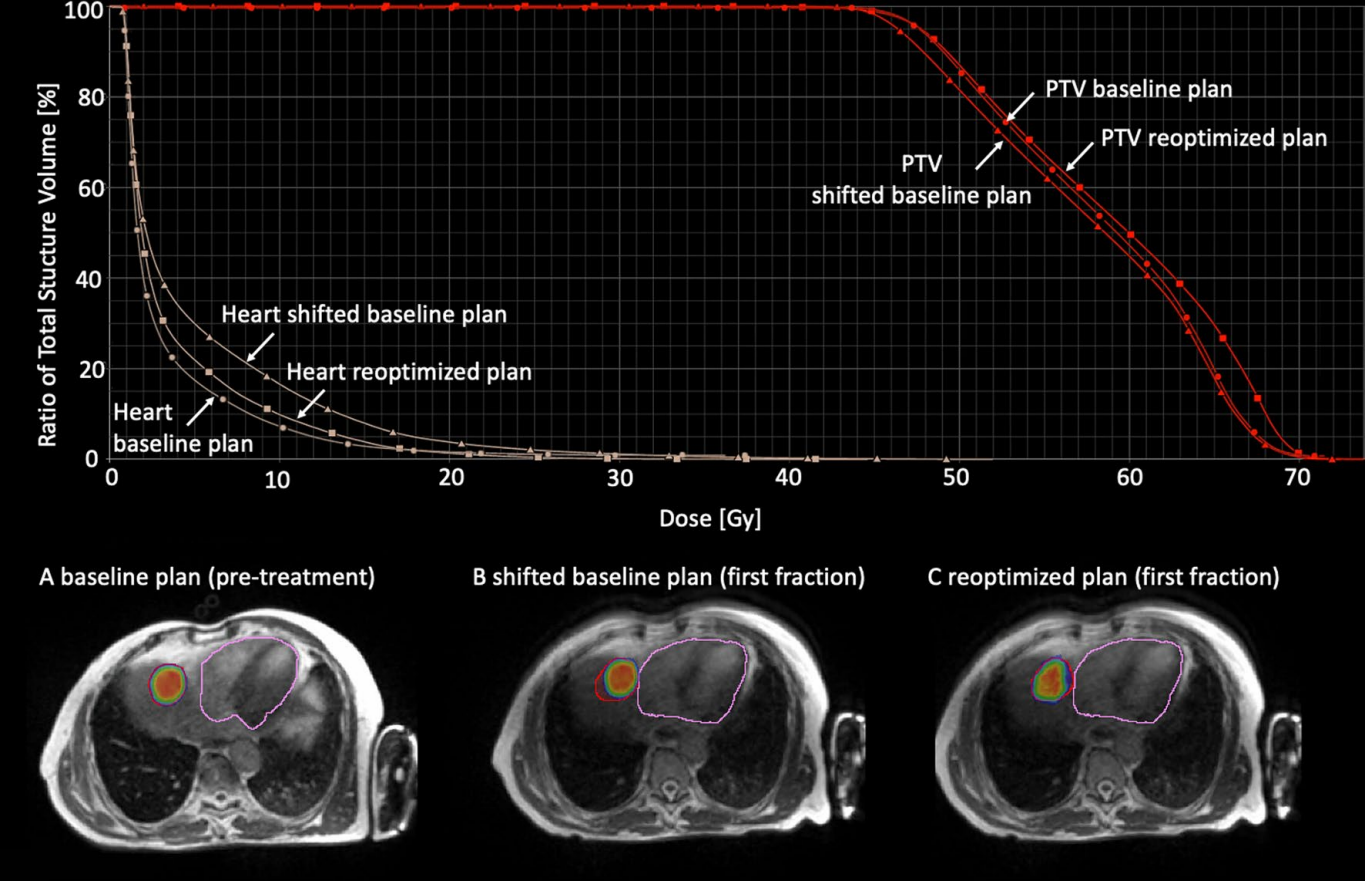
Illustration of the benefit of reoptimization for patient A: (TOP) DVH comparison of baseline, rigidly shifted, and reoptimized plan; **a** Dose distribution of the baseline plan overlayed on the pre-treatment MR; **b** rigidly shifted plan overlayed on the MR of the first treatment fraction; **c** reoptimized plan for the first treatment fraction. Doses exceeding 45 Gy are shown. The contours displayed are the PTV (red) and the heart (rose)

**Fig. 4:**
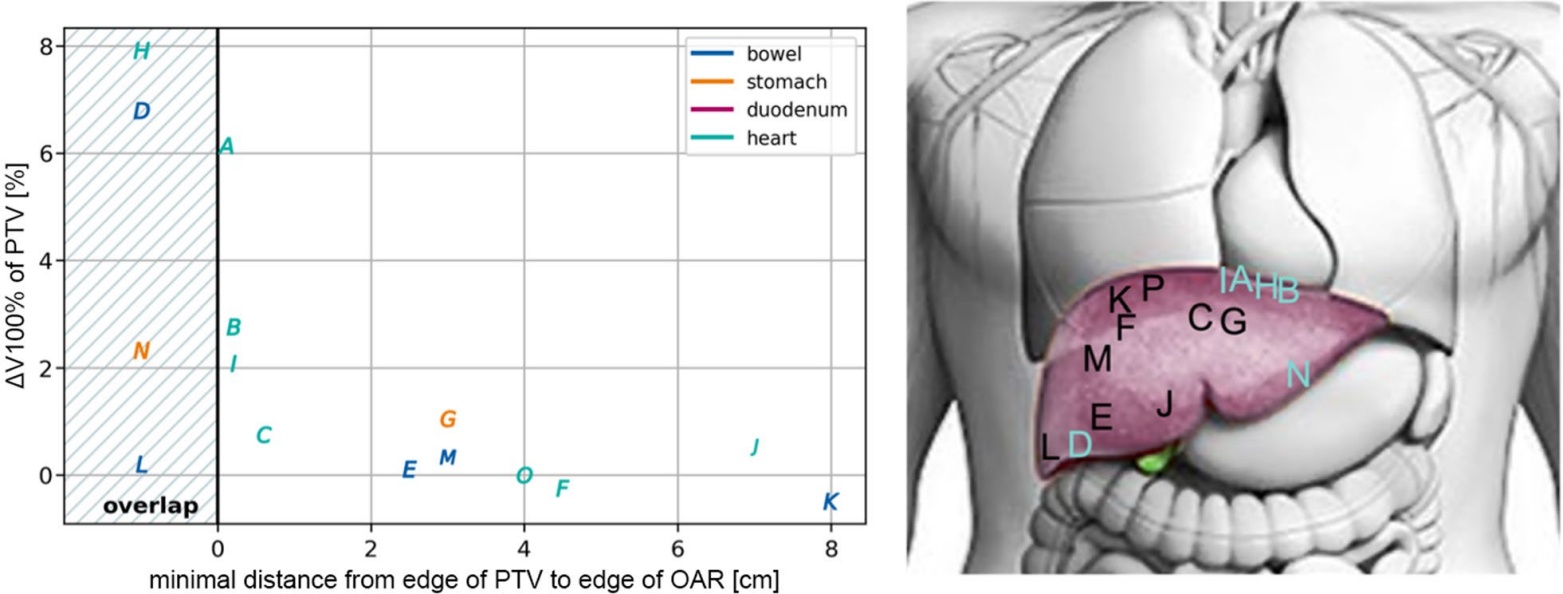
**a** Benefit of reoptimization, measured as improvement in ∆V100% as a function of the distance to the closest OAR; **b** Location of metastasis in liver. Patients with a benefit of adaptation of ∆V100% > 1% are highlighted in cyan

Compared to the sBP, RP showed improved PTV V100% and V95% coverage in 47 (63%) and 45 (60%) of the applied fractions, respectively (Figs. 2, 3). RP improved CTV V100% and GTV V100% for 18 (24%) and 8 fractions (11%) respectively when compared to sBP. Treatment adaptation significantly improved PTV V100% coverage for metastases located within close proximity of an OAR (≤ 0.2 cm distance from the edge of the PTV to the edge of the OAR; n = 7; *p* = 0.01) by 4.0 % (Fig. 4). For metastases distant from an OAR (> 2 cm; n = 7) PTV V100% coverage was not significantly improved (0.2% higher; *p* = 0.37).

Patients with OAR in close proximity were A–D, H, I, N and L. The benefit regarding ΔPTV V100_%_ for patients A–D, H, I and N is clearly visible. For patient L, ΔPTV V100% was only 0.2% higher. Nonetheless, this patient benefited from a daily online RP by a reduced total bowel dose of 4.9 Gy (sBP: 32.0 Gy vs. RP: 27.1 Gy).

RP achieved lower or maintained equal doses (for both D1cc and Dmean) in the nearest OAR in 39 of the applied 75 fractions (Fig. 2). The distance to the closest OAR for each patient is shown in Table 2.

This dosimetric effect of online replanning is illustrated in Fig. 3 showing the DVH and dose distribution for patient A for the BP and the first treatment fraction for the sBP and the RP. While the BP was of good quality (3a), the sBP was degraded as a rigid shift could not account for the altered OAR (heart) location (3b), resulting in higher cardiac dose and a decreased PTV coverage (Fig. 2). This could be solved generating a RP by a full reoptimization of the BP (3c).

### Local tumor control and toxicity

One patient, who suffered from a local relapse 7 months after treatment, was successfully treated with salvage SMART (5 × 7 Gy to the 65% isodose; salvage treatment not included in the present analysis). A diminished appetite grade I (CTCAE Version 5.0) was reported for 1 patient, while 2 patients indicated fatigue grade I. Prophylactic antiemetic medication was prescribed for 5 out of 15 patients. Three patients reported a temporary nausea grade I-II. No grade 3 treatment-related acute toxicities were observed.

With the limited median follow-up of 14 months (range 3–9 months), no late toxicities were observed.

## Discussion

While previous studies have shown a benefit of MR-based image guidance and gating for pulmonary and abdominal malignancies [13, 16–18], it is still an open question whether daily treatment plan adaptation and reoptimization is truly beneficial for all patients. As online treatment reoptimization not only entails time burden for the radiation oncologist, physicist, and therapist, but also prolongs patient-on-table time by around 30 min, a prediction of whether a particular patient might profit from daily on-table adaptive replanning instead of delivering the shifted baseline plan could significantly impact MRgRT processes. We therefore investigated whether and in which patients SMART may provide a dosimetric benefit by comprehensive DVH analysis of baseline treatment plans after rigid setup correction without re-optimization versus daily adapted plans—overlaid on the anatomy-of-the-day—on a per patient basis.

The present analysis showed that daily on-table adaptive replanning in patients with liver metastases improved PTV coverage in 63% of the applied fractions compared to a rigid shift. Previous studies have reported similar findings for patients with abdominal malignancies, where daily on-table adaptive replanning MRgRT increased PTV coverage in approximately 66% of all fractions [13, 18]. For pulmonary malignancies, adaptive treatment has been reported to improve PTV coverage in 61% of fractions [16]. These previous studies did, however, not analyze if daily on-table adaptive replanning is necessary in all patients or can safely be omitted in a specific cohort.

The benefit of treatment adaptation on PTV coverage was higher for patients with a metastasis in close proximity to an OAR compared to patients, where the GTV was at a large distance to the OARs [in Fig. 4]. The increased benefit for patients with a metastasis in close proximity to an OAR may be caused by daily positional changes of OAR, such as bowel filling and movement, by daily set-up changes. With a limited number of data points between 0.2 and 2 cm distance of OAR to PTV, the present recommendation for daily adaptive re-planning for a patient cohort with a distance of < 2 cm of the PTV to the OAR may well be too conservative but seems reasonable and feasible.

As the observed median differences for GTV and PTV volumes after plan adaptation in comparison to the BP were 0.0 cc and 0.4 cc respectively, these can be regarded as negligible. These slight variations in PTV volume were most probably caused by anatomical alterations leading to an altered CTV volume and/or inter-observer variability. As the Viewray planning software does not include the possibility to rotate a contoured structure in case of patient rotations, recontouring in some slices may also lead to slight alterations. The volume of the GTV did not change from the BP to the RP in 26/75 (34%) of all fractions. Only 7 of these 26 fractions (27%) corresponded to situations where the PTV was more than 2 cm away from the OAR. This indicates that GTV recontouring was not dependent on its proximity to the OAR. While PTV size did not change much between fractions (all volumes shown in Additional file 1), its geometry may still undergo alteration as e.g. shown in Fig. 3. These alterations may be strongly influenced by the OAR in close proximity. This is a possible explanation as to why the PTV coverage is significantly improved by MR-guided online replanning on top of MR-guided setup correction for liver metastases in close proximity to an OAR.

While improving PTV coverage, online adaptation furthermore achieved lower or maintained equal doses in OARs (D1cc and Dmean) for 54% of the applied fractions. Henke et al. reported that daily adaption could allow OAR violations to be successfully reversed in all plans, naming the primary purpose of adaption reversing OAR constraint violation in 75% of cases [13]. The constraints of the trial by Henke et al. were, however, less conservative than the ones employed in the present study and this could explain the observed difference.

The present analysis is limited by its small sample size and use of different fractionation schemes. Intra-fraction motion could lead to altered OAR and GTV doses. In such cases, a post-treatment scan may add additional information concerning the dose distribution. While continuous sagittal cine MR-imaging and tracking of the GTV was performed in the present study, future investigations may benefit from additional post-treatment imaging. While the required time for online adaptation exceeds durations for typical SBRT fractions, it corresponds to procedures such as robotic SBRT or brachytherapy [19, 20]. With the advent of technical advancements, such as automated adaption, future treatment times for SMART could even be reduced considerably [21]. Therefore, this study results may not be as relevant in the future as now, when treatment times will be significantly reduced. However, currently every effort to reduce slot time is relevant to provide sufficient machine time to treat all patients suitable for MRgRT.

## Conclusions

MR-guided online replanning SBRT on top of MR-guided setup correction and gating of liver metastases resulted in improved target coverage and OAR sparing for liver metastases with a distance of < 0.2 cm from the edge of the PTV to the nearest luminal OAR. Only marginal improvements in target coverage were observed for target distant to critical OARs, indicating that these patients do not benefit from daily adaptive replanning.

## Supplementary Information

**Additional file 1**. Adaptive volume changes of GTV and PTV.

## Data Availability

The datasets used and/or analysed during the current study are available from the corresponding author on reasonable request.
